# Effects of Serving as a State Functionary on Self-Rated Health: Empirical Evidence From China

**DOI:** 10.3389/fpubh.2022.757036

**Published:** 2022-04-01

**Authors:** Li He, Zixian Zhang, Jiangyin Wang, Yuting Wang, Tianyang Li, Tianyi Yang, Tianlan Liu, Yuanyang Wu, Shuo Zhang, Siqing Zhang, Hualei Yang, Kun Wang

**Affiliations:** ^1^School of Philosophy, Zhongnan University of Economics and Law, Wuhan, China; ^2^School of Public Administration, Zhongnan University of Economics and Law, Wuhan, China

**Keywords:** state functionary, civil servants, occupation, self-rated health, China Labour-Force Dynamics Survey

## Abstract

**Purpose:**

There is a strong link between occupation and self-rated health. Existing research has revealed the effects of occupation on self-rated health outcomes and the corresponding mechanisms. However, there is a lack of research on the effects of state services on self-rated health in China. Therefore, this study focuses on exploring the effects of serving as a state functionary in China on self-rated health to enrich research in related fields.

**Method:**

Based on the data of 14,138 individuals collected from the 2016 China Labour-Force Dynamics Survey, the logit model was used to investigate the effects of serving as a state functionary on self-rated health and the difference in the effects across different populations.

**Results:**

The results show that (1) serving as a state functionary has a significant positive effect on self-rated health; (2) self-rated health of elderly state functionaries is higher than that of younger state functionaries; (3) self-rated health of state functionaries in non-eastern regions is higher than that of state functionaries in eastern regions; and (4) state functionaries with lower education have higher self-rated health than highly-educated state functionaries; (5) Higher self-rated health of state functionaries is achieved primarily through better work time, better work environment and lower relative deprivation.

**Conclusion:**

Serving as a state functionary in China has a significant positive correlation with self-rated health, with differences across populations of state functionaries. This study expands the current literature on the effects of occupation on self-rated health in the context of China.

## Introduction

State functionary refers to any person engaged in public service in party and government offices, public institutions, mass organizations, or enterprises. The 2019 Green Book on the Health of China's Civil Servants, based on big data from the physical examination of 3,00,000 civil servants, reported that the detection rate of hypertension, hyperlipidaemia, hyperglycaemia, and hyperuricemia among civil servants was higher than the average level in Chinese adults.^1^ In general, due to the particularity of their occupations and the realistic environment in China, the incidence of chronic diseases among state functionaries is high, and their health is poor. It raises the question of whether state functionaries are in worse health than other workers. From a theoretical point of view, the answer to this question enriches and complements the field of occupational and health research, especially by linking specific occupational types with self-rated health at the micro level. From the perspective of reality, this study is not only beneficial for the state to adjust and formulate relevant policies on occupational security and social welfare but can also provide the public with a clearer occupational reference, which is conducive to job seekers to make rational choices.

Self-rated health is a comprehensive measure of the general health status of individuals perceived by themselves. It reflects the overall perception of people's self-health and can be regarded as an indicator of the incidence and mortality rates of the general population. It is an important indicator in health research (1, 2). The factors that influence self-rated health are diverse. Occupation, a means for people to make a living, plays an important role. In research on the relationship between occupation and self-rated health, scholars worldwide have made many useful explorations. Specifically, the literature is divided into two modules: the relationship between occupation and self-rated health and the influencing factors behind them. This has done essential theory foreshadowing for this study to further explore the relationship between state functionaries and self-rated health.

First, regarding the relationship between occupation and self-rated health, there are some conflicting conclusions in the literature. Some scholars have observed that white-collar workers have substantially better self-rated health than other occupational groups (1). Blue-collar workers such as farmers, foresters, fishing workers, handlers, equipment cleaners, laborers, protective service workers, private household workers, machine operators, assemblers, and inspectors are more likely to report poor self-rated health (3, 4). In a survey of the working population in central and western France, Murcia et al. (5) observed a strong social gradient in self-rated health and concluded that low occupational groups, especially manual and service workers, were more likely to report poor health. Even for non-manual workers, the odds of reporting less than good self-rated health increases with each added year of previous manual experience (6). In subsequent studies of the working population in 27 European countries and Brazil, the researchers confirmed that low occupational groups were associated with poorer self-rated health (2, 7). Although most scholars have found that the self-rated health of low occupational groups such as service personnel and manual workers is often very poor, some scholars' research results partially deny this conclusion (8). Those scholars identified that personal care and service employees had the highest prevalence of fair or poor self-rated health. Additionally, they found that legal, business, and financial operators had a high prevalence of fair or poor self-rated health, whereas farmers, fishing workers, and forestry employees had the lowest prevalence of fair or poor self-rated health, contradicting previous findings.

Second, the relationship between occupation and self-rated health is regulated by other factors. It has been found that gender, education level, income level, social professional class, working environment, work pressure, and other factors have a certain effect on self-rated health (9–11). It is widely recognized that education and income levels can have an effect on self-rated health, but whether education level is positively related to health is still uncertain. Some studies have pointed out that people in low educational and income groups have worse self-rated health status (12, 13). Some recognized a trend toward lower age-specific self-rated health at all levels of education, but less so the higher the education (14). As for the occupational social class, the results show a marked social gradient in self-rated health. The prevalence of poor or moderate health in men and women under 50 years of age in manual classes is similar to that seen in men and women over 70 years old in non-manual classes; men and women in unskilled occupations are approximately twice as likely to report poor or moderate subjective health as those in professional occupations; the probability of declaring themselves healthy is greater for agricultural classes than for non-agricultural classes (15, 16). In terms of work environment factors, repetitive work, high psychological demands, low social support, high job insecurity, and high ergonomic exposure all have more effects on the low self-rated health index (17). Additionally, there is strong evidence of an association between job strain and adverse health outcomes, including coronary heart disease and associated risk factors, musculoskeletal pain, and psychological ill-health (7). Furthermore, some scholars believe that long working hours, shift work, and night shifts can lead to poor self-rated health, including sleep deprivation, depression and anxiety disorders, and cardiovascular diseases, especially stroke (18). However, other scholars did not find a strong link between long working hours and self-rated health (2).

This study focuses on examining the effects of being a state functionary in China on self-rated health. Moreover, this study investigates the heterogeneity of this effect by age, region, and educational background and tests the applicability of existing empirical findings to Chinese samples.

## Materials and Methods

### Data

The data were extracted from the 2016 China Labour-Force Dynamics Survey. The survey adopts a multi-stage, multi-level, probability sampling method that is proportional to the size of the labor force and is the first to adopt a rotating sample tracking method, which can better adapt to China's drastically changing environment. At the same time, the survey involves the content of population, economy, society, psychology, health, and other multidisciplinary fields and provides large samples and scientific and objective social science data for multidisciplinary or interdisciplinary research. Considering the applicability of the problem, this study restricted the research object to the labor force working since January 2015. After excluding missing values and illogical responses, 14,138 samples were analyzed.

### Variable

#### Self-Rated Health

This study considered self-rated health as an explained variable. Empirical research on China shows that self-rated health presents good reliability and validity and is a comprehensive indicator that reflects individual health status. To more accurately reflect the effects of being state functionaries on self-rated health, this study defined that individuals who self-rated themselves “very healthy”, “relatively healthy”, or “average” were in a healthy state, with a value of 1, and individuals with “relatively unhealthy” or “very unhealthy” self-health assessment were in an unhealthy state and were assigned a value of 0.

#### State Functionary

In China, *bianzhi* is the fundamental criterion for determining whether an individual is a state functionary. *Bianzhi* is the system of determining the number of personnel to be appointed by government agencies, public institutions and other organizations (19). The *bianzhi* has a strong state function attribute and professional stability, which is a unique phenomenon in China. In general, only state functionaries have *bianzhi*. Therefore, this study defined the sample with *bianzhi* as state functionaries and assigned them a value of 1, and the sample without *bianzhi* was a non-state functionary with a value of 0.

#### Control Variables

Based on research questions, considering the factors that affect workers' self-evaluation of health, and referring to the research of Marchand et al. (20), this study selected sex, age, education, marital status, religious belief, social interaction, life choice, income, and region as control variables. The definitions of variables and descriptive statistics are presented in Table 1.

**Table 1:** Variable definition and descriptive statistics.

**Variable**	**Definition**	**Observations**	**Mean**	**Std. Dev**.	**Min**	**Max**
Self-rated health	Very healthy, relatively healthy, average = 1; relatively unhealthy, very unhealthy = 0	14,138	0.870	0.337	0	1
Occupation	State functionary = 1,otherwise = 0	14,138	0.074	0.261	0	1
Sex	Male = 1,female = 0	14,138	0.536	0.499	0	1
Age	Actual age of the respondent	14,138	45.708	13.001	15	96
Education	Assign value from low to high	14,138	8.689	4.252	0	23
Marital status	First marriage, remarriage = 1; unmarried, divorced, widowed, cohabitation = 0	14,138	0.869	0.338	0	1
Religious belief	Yes = 1, no = 0	14,138	0.127	0.333	0	1
Social interaction	Very unfamiliar = 1, not very familiar = 2, general = 3, relatively familiar = 4, very familiar = 5	14,138	3.814	0.99	1	5
Life choice	The degree of choice in life^a^	14,138	6.801	2.174	1	10
Income	Respondent's personal total income last year, transformed by the logarithm	14,138	9.232	2.552	0	14.931
Region	Eastern region = 1, other regions = 0	14,138	0.425	0.451	0	1

a *The score ranged from 1–10; the higher the score, the greater the degree of life choice*.

### Model

The explanatory variable in this study was a binary variable, which cannot meet the continuous explained variable assumption of the ordinary linear regression model. Therefore, this study adopted a logit model to examine the effects of being a state functionary on self-rated health. The model was defined as follows:


(1) $$\begin{array}{r} health_{i}=\alpha_{0}+\alpha_{1}occupation_{i}+\alpha_{2}X_{i}+\varepsilon_{i} \end{array}$$

where *health*_*i*_ represents self-rated health status of the i-th respondent; *occupation*_*i*_ represents whether the i-th respondent was a state functionary; *X*_*i*_ refers to other control variables; ε_*i*_ represents the random error term; and α_1_is the coefficient to be estimated, which reflects the direction and magnitude of the influence of being state functionaries on self-rated health.

To address the endogeneity problem caused by self-selection, this study employed the propensity score matching method proposed by Rosenbaum and Rubin (21). Specifically, we first calculated the conditional probability of an individual serving as a state functionary, that is, the propensity score (PS).


(2) $$\begin{array}{r} \text{PS}\left(\text{X}\right)=\text{Pr}\left\{\text{D}=1|\text{X}\right\}=\text{E}\left\{\text{D}|\text{X}\right\} \end{array}$$

where *D* is a dummy variable for whether the individual is a state functionary. If the individual is a state functionary, *D* = 1; otherwise *D* = 0. X are the covariates that affect whether an individual is a state functionary. Drawing lessons from the research ideas of Dehejia and Wahba (22), this study defined the following logit model:


(3) $$\begin{array}{r} \text{PS}\left(\text{Xi}\right)=\text{Pr}\left\{\text{Di}=1|\text{Xi}\right\}=\text{exp}\left(\text{βXi}\right)/\left(1+\text{exp}\left(\text{βXi}\right)\right) \end{array}$$

where β is the regression coefficient and PS is the predicted value of the logit model.

We next matched the treatment group with the control group according to PS values and searched for individuals with the smallest absolute difference in PS for pairing. After the matching, the control group only retained the individuals with characteristics closest to the treatment group and eliminated all other individuals. Finally, in the matched samples, we used probit and logit models to explore the effects of being a state functionary on self-rated health.

In addition, considering the possible reverse causality between serving as a state functionary and self-rated health, we further used the instrumental variable to address the endogeneity problem. The selection of instrumental variable should meet two basic conditions: first, the instrumental variable is highly correlated with the endogenous explanatory variable; Second, the instrumental variable has no direct impact on the explained variable. Based on these, this study took the proportion of state functionaries in the city where the respondents belong as the instrumental variable of individual serving as a state functionary. Its rationality lies in: (1) the higher the proportion of state functionaries in the city, the more likely individuals are to serve as state functionaries, meeting the requirement of high correlation of instrumental variable; (2) There is no enough evidence that the proportion of state functionaries in the city to which they belong affects their health.

## Results

### Benchmark Regression

Table 2 presents the results of benchmark regression analysis. Model 1 is an ordinal logit regression, and Model 2 is a logit regression. Self-rated health in Model 1 ranged from 1 to 5. The higher the score, the worse the self-rated health. In Model 2, self-rated health had a binary outcome, with 1 being healthy and 0 being unhealthy. The interpretation of the results is based on Model 2. Model 1 in Table 2 shows that the odds ratio of self-rated health in state functionaries is 11.3% lower than that in other staff. This indicates that serving as a state functionary significantly improves self-rated health. Table 2, Model 2 shows that the odds ratio of self-rated health in state functionaries is 42% higher than that in other staff and is significant at the 5% level. This indicates that serving as a state functionary has a significant positive effect on self-rated health.

**Table 2:** Results of benchmark regression analysis.

	**Model 1**	**Model 2**
Occupation	0.887^**^	1.420^**^
	(0.052)	(0.227)
Sex	0.843^***^	1.196^***^
	(0.027)	(0.068)
Age	1.041^***^	0.955^***^
	(0.002)	(0.002)
Education	0.958^***^	1.085^***^
	(0.005)	(0.008)
Marital status	0.885^**^	1.040
	(0.04)	(0.100)
Religious belief	0.866^***^	1.099
	(0.041)	(0.091)
Social interaction	0.844^***^	1.046
	(0.015)	(0.031)
Life choice	0.894^***^	1.092^***^
	(0.007)	(0.014)
Income	0.929^***^	1.100^***^
	(0.006)	(0.010)
Region	0.859^***^	1.502^***^
	(0.027)	(0.085)
_cons		5.287^***^
		(1.120)
*N*	14138	14138
Pseudo-R^2^	0.053	0.128

** *p < 0.05*.

*** *p < 0.01*.

*Odds ratio is reported. Robust standard errors are indicated in parentheses*.

Regarding other control variables, men had significantly better self-rated health than women. Self-rated health gradually deteriorated with age. Compared with low-educated people, those with a high level of education had significantly better self-rated health. People with high life choices had higher self-rated health. Higher income is associated with higher self-rated health. People in the eastern region^2^ self-assessed their health better than those in other regions.

### Endogenous Analysis

Table 3 reports the results of the two-stage least square regression after using the instrumental variable. Model 3 is the first-stage regression result, from which it can be seen that the regression coefficient of the endogenous explanatory variable on the instrumental variable is significant, and the null hypothesis is rejected at the 1% level. This shows that the instrumental variable is strongly correlated with the endogenous explanatory variable. In addition, the statistic value of *F* in the first stage is >10, dismissing possibility of weak instrumental variable. Model 4 is the regression result of the second stage. It can be seen that the regression coefficient of the potential endogenous explanatory variable is significant at the 1% level, indicating that being a state functionary can significantly improve self-rated health. This is consistent with the results estimated in the benchmark regression and proves the positive impact of being a state functionary on self-rated health.

**Table 3:** Instrumental variable method: 2SLS.

	**Model 3**	**Model 4**
IV	0.274^***^	
	(0.014)	
occupation		0.375^***^
		(0.070)
Control variables	yes	yes
*F*	398	
*N*	14138	14138
*R*2	0.114	0.030

*** *p < 0.01*.

### Robustness Test

To ensure the reliability of the results, this study further used propensity value matching. We first checked the validity of the propensity value matching method. Table 4 presents the results of the balance tests. All covariates have deviations <10% after matching, and the *P*-value is not significant, which proves that all covariates are balanced. Table 5 shows the results of regression using the matched samples. Model 5 is a probit model, and Model 6 is a logit model. The regression results of the two Models are consistent with the original findings, proving their reliability.

**Table 4:** Results of balance test.

**Variable**	**Match**	**Mean**		**Bias (%)**	**Reduced**	***t*-value**	***p*-value**
		**Treated**	**Control**		**Bias (%)**		
sex	*U*	0.622	0.542	16.2		4.81	0.000
	*M*	0.623	0.618	1.0	94.1	0.21	0.831
age	*U*	42.709	45.891	−27.9		−7.49	0.000
	*M*	42.716	43.044	−2.9	89.7	−0.66	0.511
education	*U*	13.967	8.326	161.7		42.80	0.000
	*M*	13.958	13.908	1.4	99.1	0.38	0.701
marital status	*U*	0.865	0.871	−1.9		−0.56	0.573
	*M*	0.865	0.86	1.5	21.9	0.32	0.753
social interaction	*U*	3.434	3.846	−41.0		−12.56	0.000
	*M*	3.436	3.395	4.0	90.1	0.86	0.392
life choice	*U*	6.395	6.864	−22.4		−6.55	0.000
	*M*	6.396	6.317	3.8	83.2	0.84	0.402
smoke	*U*	0.333	0.333	0.1		0.03	0.978
	*M*	0.333	0.336	−0.6	−589.4	−0.14	0.891
drink	*U*	0.279	0.237	9.6		2.96	0.003
	*M*	0.279	0.289	−2.2	77.6	−0.46	0.644
exercise	*U*	0.543	0.264	59.3		18.83	0.000
	*M*	0.544	0.556	−2.5	95.8	−0.51	0.607
region	*U*	0.403	0.424	−4.3		−1.28	0.201
	*M*	0.403	0.406	−0.6	86.9	−0.12	0.902

*U, unmatched, M, matched*.

**Table 5:** Regression results after matching.

	**Model 5**	**Model 6**
occupation	0.160^*^	1.422^**^
	(0.084)	(0.250)
Control variables	yes	yes
*N*	2,973	2,973
r2/pr2	0.114	0.105

* *p < 0.1*.

** *p < 0.05*.

*Regression coefficient in model 5, odds ratio in model 6. Robust standard errors are indicated in parentheses*.

In addition, we used two other different methods to measure the health status of the respondents to ensure the robustness of our results. Specifically, first, when self-rated health was transformed into a dummy variable, the average response was in an unhealthy state rather a healthy state; Second, the frequency of feeling physical pain in the past month was used to represent the individual health status, with a value of 1 for those who felt pain and 0 for those who did not feel pain. Table 6 reports the results of the health impact of serving as a state functionary under different health measures. Model 7 is the result of changing the self-rated health assignment, and Model 8 is the result of pain frequency representing health. It is found that being a state functionary still has a significant positive effect on self-rated health after the change of self-rated health values. The same result is also found when pain is used as the proxy for individual health. State functionaries are less likely to feel physical pain than non-state functionaries, indicating that they are at a higher level of health status.

**Table 6:** Regression results after changing health measures.

	**Model 7**	**Model 8**
occupation	1.200^***^	0.699^**^
	(0.093)	(0.105)
Control variables	yes	yes
*N*	14,138	14,138
Pseudo-R2	0.080	0.105

** *p < 0.05*.

*** *p < 0.01*.

*Odds ratio is reported. Robust standard errors are indicated in parentheses*.

### Mechanism Analysis

The above empirical results show that being a state functionary has a positive impact on self-rated health. This section continues to explore the mechanisms by which this effect is achieved. Referring to existing studies (23–25), we mainly considered three mediating mechanisms: work time, work environment and relative deprivation. Work time is measured by the individual satisfaction with working hours, and is rated from 1 to 5. The higher the value, the more satisfied they are with their work schedule. The work environment is measured by the individual satisfaction with the work environment, which is assigned as a 5-point Likert scale, 1= very dissatisfied, 5= very satisfied. Relative deprivation is measured by individual attitude toward the relationship between their work reward and effort. The value ranges from 1 to 5. The higher the value is, the weaker the sense of relative deprivation is.

The results of the mediating effect analysis are reported in Table 7. Models 9, 11, and 13 are the regression results of mediating variables to independent variables; Models 10, 12, and 14 are the regression results with each mediating variable added separately to the benchmark regression. It can be seen from Model 9 that state functionaries have higher satisfaction with their work time. The results of Model 10 show that the odds ratio of self-rated health of state functionaries decreased after adding the mediating variable of work time, and both the mediating variable and the core explanatory variable are significantly correlated with self-rated health. This suggests that work time is one of the mediators of higher self-rated health among state functionaries. In the results of Models 11, 12, 13, and 14, we found that work environment and relative deprivation are also mediators of higher self-rated health among state functionaries.

**Table 7:** The results of the mediating effect analysis.

	**Model 9**	**Model 10**	**Model 11**	**Model 12**	**Model 13**	**Model 14**
	**Work time**	**Health**	**Work environment**	**Health**	**Relative deprivation**	**Health**
Occupation	0.159^***^	1.370^**^	0.092^***^	1.391^**^	0.120^***^	1.372^*^
	(0.029)	(0.220)	(0.028)	(0.222)	(0.031)	(0.221)
Work time		1.141^***^				
		(0.035)				
Work environment				1.192^***^		
				(0.039)		
Relative deprivation						1.406^***^
						(0.041)
CONTROL variable	yes	yes	yes	yes	yes	yes
*N*	13,767	13,767	13,815	13,815	14,138	14,138
r2/pr2	0.067	0.129	0.058	0.129	0.058	0.141

* *p < 0.1*.

** *p < 0.05*.

*** *p < 0.01*.

*Regression coefficient in models 9, 11, 13, odds ratio in models 10, 12, 14. Robust standard errors are indicated in parentheses*.

### Heterogeneity Analysis

This study next examined whether the effects of being a state functionary on self-rated health differs by age, region, and educational background. The results of the heterogeneity analysis are presented in Table 8. Model 15 shows that elderly state functionaries have better self-rated health than younger state functionaries. Model 16 indicates that state functionaries in non-eastern regions have better self-rated health than those in the eastern region. Model 17 reveals that state functionaries who have not gone to college have higher self-rated health than those who have gone to college.

**Table 8:** Results of heterogeneity analysis.

	**Model 15**		**Model 16**		**Model 17**	
	**Age ≤35**	**Age > 35**	**Eastern region**	**Other regions**	**Have studied at college**	**Never attended college**
occupation	0.561	1.683^***^	0.842	1.999^***^	0.750	1.403^***^
	(0.244)	(0.289)	(0.192)	(0.447)	(0.336)	(0.254)
Control variables	yes	yes	yes	yes	yes	yes
*N*	3463	10675	6002	8136	959	13179
Pseudo-*R*^2^	0.050	0.089	0.125	0.117	0.118	0.119

*** *p < 0.01*.

*Odds ratio is reported. Robust standard errors are indicated in parentheses*.

### Further Analysis

To examine differences in self-rated health between state functionaries and other professionals, we further analyzed the occupational sub-sample (Table 9). According to the classification of occupation types in the China Labour-Force Dynamics Survey, further comparisons were made between state functionaries and those working in “autonomous organizations”, “private enterprises”, “social organizations”, and as “peasantries”, and “freelancers”. Regression analysis was performed for each sub-sample, and it was found that the self-rated health of state functionaries was higher than that of other workers, especially peasants.

**Table 9:** Results of further analysis.

	**Full sample**	**Autonomous organizations**	**Private enterprises**	**Social organizations**	**Peasantries**	**Freelancers**
Occupation	1.420^***^	1.872	1.318	1.712	2.698^***^	1.543^*^
	(0.227)	(0.998)	(0.389)	(1.047)	(0.451)	(0.350)
Control variables	yes	yes	yes	yes	yes	yes
*N*	14,138	1,312	3,804	1,104	7,270	2,359
Pseudo-*R*^2^	0.128	0.157	0.136	0.167	0.122	0.139

* *p < 0.1*.

*** *p < 0.01*.

*Odds ratio is reported. Robust standard errors are indicated in parentheses*.

## Discussion

### State Functionaries Have Higher Levels of Self-Rated Health than Non-state Functionaries

Compared with non-state functionaries, Chinese state functionaries rate their health more highly in response to better working conditions and the availability of medical services. State functionaries enjoy a higher level of medical security. Whether medical security can meet health needs has a great effect on the health of individuals, and the lack of access to medical services can negatively affect self-rated health (26). State functionaries receive better medical insurance benefits, higher public health subsidies, and undergo regular checkups. They are better able to identify health problems timely and are willing to access more medical resources to address them. Because state functionaries have access to more medical support and services and are more aware of their own health conditions, they rate their health more highly.

The working conditions of state functionaries are better than non-state functionaries. First, in terms of work content, most state functionaries are engaged in mental work, which is less harmful to the body. A large proportion of non-state functionaries, especially manual workers, tend to suffer from some form of physical injury. Second, state functionaries have ideal working hours. Compared to non-state functionaries, state functionaries have a more scientific work-rest allocation and are less likely to experience long working hours and overwork. Finally, the working environment of state functionaries is superior. Non-state functionaries, especially low occupational classes, have poor working environments. For example, manual workers are more likely to be exposed to harsh working environments and long-term negative effects of irritating chemicals, dust, high temperature, physical exertion, and other factors and are at greater risk of work-related injuries and chronic diseases (27). As a result, manual workers are more likely to report poor health (5, 28). In general, better working conditions make state functionaries have a lower risk of health problems, and they rate their health status more highly than non-state functionaries.

### Older State Functionaries Have Higher Levels of Self-Rated Health Than Younger Ones

In China, older age means higher position, rank, and social status, and older people, in general, have more life experience than younger people. Differences in social status and life experience lead to differences in health status, health attention, and compressive strength.

Elderly state functionaries in China with higher social status and rank generally have better health. China's state system has two main characteristics. First, employment is usually for life, especially for those who enter the state system early. As long as employees do not quit or make mistakes, they will usually not be fired, which ensures job stability and security. Second, promotion, salary, and benefits are based on seniority. In other words, the older a state functionary is, the more likely it is that he or she is promoted, and the higher the salary and benefits. Studies have found that higher position and better economic status are generally associated with better health status (29), which also leads to a higher evaluation of self-health.

Furthermore, because of their rich life and work experience, older state functionaries are better able to cope with stress. They have been engaged in work for much longer than young people, have experienced more accidents and emergencies at work, have more problem-solving experience, and have a better mentality (30). In other words, aging can be seen as a psychological advantage, giving the elderly a better ability to cope with stress (31, 32). This ability to resist stress can effectively alleviate the negative effects of stress on the body, resulting in a higher level of self-rated health. However, the sense of mastery of young people to control of their stress and work weaker than older people (33) and are extremely vulnerable to pressure from “work overload” or emergencies. Being exposed to continuous stress has a negative effect on the body and mind, making it harder for young people to engage in desirable health-related activities, which leads to lower self-rated health.

### State Functionaries in Non-eastern Regions Have Higher Levels of Self-Rated Health than Those in Eastern Regions

The eastern regions in China are more economically developed than other regions. As a result, the gap between pay, benefits, and working conditions of state functionaries and other workers is relatively small. Additionally, people in economically developed areas are more sensitive and demanding of public services, which puts more pressure on state functionaries.

In general, economic development is often accompanied by an increase in living costs. A higher cost of living results in less disposable income for health protection. The cost of living is higher in the eastern regions, but with the same basic salary across the country, state functionaries in the eastern regions have less surplus disposable income and spend less on health security. Adequate health protection improves individuals' self-rated health levels. The more medical security individuals seek, the higher their self-rated health (26). Because of the lower cost of living in non-eastern regions, state functionaries in these regions are more likely to spend more of their income on health protection, are more likely to be proactive, and have higher self-rated health levels.

Competitive work positions and higher work stress are strongly associated with a higher likelihood of reporting “poor health” (7). In general, areas with higher levels of economic and social development have more complicated public affairs. As a result, state functionaries in the eastern regions are faced with more work tasks, higher work requirements, and more fierce competition and are under relatively higher pressure. The high intensity of work and the resulting high stress make state functionaries in the eastern regions more prone to health problems, which leads to them having relatively low self-rated health levels. In contrast, state functionaries from non-eastern regions, who have relatively few tasks, suffer less stress and have higher levels of self-rated health.

### State Functionaries With Lower Education Have Higher Levels of Self-Rated Health Than Those With Higher Education

Educational background is an important factor affecting the self-rated health of state functionaries. Educational background is closely related to job promotion, work energy input, and the initial age of work among state functionaries.

First, when entering the state functionary system, people with both low and high education receive roughly the same salary and benefits. Compared with highly-educated people, low-educated individuals invest less in human capital, and their sense of efficacy is stronger, leading to their higher self-rated health.

Second, educational background is positively correlated with promotion opportunities of state functionaries. Having a certain level of education is the first requirement for a position in the Chinese party and government agencies. Many people are hindered in their careers because they have not reached the level of education required for a position or promotion (34). Thus, education tends to increase one's job opportunities (35), and people with higher education will have more advantages in promotion. State functionaries with less education, who have less room for advancement, are more likely to give up competition. Therefore, they have less pressure to be promoted than those with higher education. This not only reduces physical and mental stress at work, but also allows them more free time to focus on their personal lives, such as their health. As a result, the levels of self-rated health of state functionaries with lower education are higher.

Third, highly educated state functionaries devote more time and energy to their work. Compared with low-educated people, highly-educated individuals tend to have higher expectations from themselves and their work and pursue high-quality work tasks. This means that they put more time and energy into work; however, a negative correlation was found between long working hours and self-rated health (36–38). Working long hours makes people more likely to feel fatigued (39), and the work stress is subjectively amplified, which leads to the deterioration of individual psychological state (40), affecting their self-rated health. Moreover, long working hours leave little time for rest. This not only increases the risk of diseases such as high blood pressure (41, 42) but also makes people less likely to engage in physical activities (43) and disease prevention, resulting in worse physical fitness and self-rated health. That is, the maintenance of health requires individuals to have enough rest time to carry out certain healthy activities and behaviors. Physical activities that lack leisure time have a significant effect on self-perceived health status (10), which leads to poor self-rated health. As a result, highly educated state functionaries have lower self-rated health.

Finally, state functionaries with low education have the advantage of a low initial age at work. Low-educated people are more adaptable to work and have a lower initial age at work. Young people are not only more malleable (44) but also have better physical fitness and are able to withstand the physical pressure brought by work. In contrast, highly-educated people are older when they enter the workforce and are less adaptable to work. Furthermore, people with lower education can accumulate more working experience as they encounter more work accidents and emergencies. Therefore, the advantages brought by the initial age of work enable state functionaries with lower education to adapt to the working environment earlier and have more work experience than those with higher education. In this way, they can spend more time focusing on their health and carrying out activities to improve their health when their work enters a stable stage. As a result, their level of self-rated health is higher.

## Conclusion

This study used data from the 2016 China Labour-Force Dynamics Survey to explore the effects of serving as a state functionary on self-rated health. The results of this study show a strong association between serving as a state functionary and self-rated health. Specifically, there was a significant positive correlation between serving as a state functionary in China and self-rated health, and the former increased the odds of self-rated health by 42%. Additionally, the self-rated health of older people serving as state functionaries was higher than that of younger state functionaries, and those in the non-eastern regions was higher than state functionaries in the eastern regions. The self-rated health of state functionaries with low education or no college education was significantly higher than that of people with high education. The mediating effect analysis found that the higher self-rated health of state functionaries was mainly achieved through better work time, better work environment and lower relative deprivation.

Based on the above findings, this study makes the following suggestions to improve workers' self-health perception: first, attention should be paid to the fairness of welfare treatment among different occupations and positions, avoiding occupational discrimination caused by the inequality of welfare treatment. Second, special attention needs to be paid to age-related health status, especially the health status of young state functionaries. The key is to change China's lifetime employment and seniority systems. Third, the high cost of living and work requirements in eastern China have worsened the health of state functionaries. Raising wages and a relatively slow working pace can reduce the negative effects of overwork on health. Fourth, the educational background should not be mandatory for state functionary recruitment; rather, the focus should be on the practical ability of workers. The health status of highly-educated state functionaries also demands attention. A more perfect promotion evaluation system would reduce the damage caused by promotion pressure on physical health.

The limitation of this research is that we use cross-sectional data rather than panel data to examine the impact of being state functionaries on self-rated health. We acknowledge that data limitations make this difficult to solve. We leave these aspects for future research.

## Data Availability Statement

The data used in this study was obtained by applying to the Center for Social Survey of Sun Yat-sen University cssdata@mail.sysu.edu.cn. Please contact the corresponding author if necessary. Email: wk123@stu.zuel.edu.cn.

## Author Contributions

LH: conceptualisation, supervision, and funding acquisition. KW: method, software, and visualisation. KW, ZZ, and JW: validation. JW: formal analysis and investigation. YWa, TY, and TLiu: resources. HY: data curation and project administration. ZZ, JW, and TLi: writing—original draft preparation. LH, YWu, ShZ, and SiZ: writing—review and editing. All authors have read and agreed to the published version of the manuscript.

## Funding

This research was funded by the National Social Science Foundation of China (Grant Number: 20CZZ012) and the Humanities and Social Sciences Fund of the Ministry of Education (Grant Number: 19YJC790167).

## Conflict of Interest

The authors declare that the research was conducted in the absence of any commercial or financial relationships that could be construed as a potential conflict of interest.

## Publisher's Note

All claims expressed in this article are solely those of the authors and do not necessarily represent those of their affiliated organizations, or those of the publisher, the editors and the reviewers. Any product that may be evaluated in this article, or claim that may be made by its manufacturer, is not guaranteed or endorsed by the publisher.

## Footnotes

^1^http://www.xinhuanet.com/health/2019-03/19/c_1124253594.htm.

^2^The eastern region includes Beijing, Tianjin, Hebei, Shandong, Jiangsu, Shanghai, Zhejiang, Fujian, Guangdong, Hainan provinces and cities.

## References

[1] Arheart KL Fleming LE Lee DJ Leblanc WG Caban-Martinez AJ Ocasio MA. Occupational vs. industry sector classification of the US workforce: Which approach is more strongly associated with worker health outcomes? Am J Ind Med. (2011) 54:748–57. 10.1002/ajim.20973 21671459 PMC3168588

[2] Oenning NSX de Goulart BNG Ziegelmann PK Chastang JF Niedhammer I. Associations between occupational factors and self-rated health in the national Brazilian working population. BMC Public Health. (2019) 19:1381. 10.1186/s12889-019-7746-5 31655583 PMC6815372

[3] Niedhammer I Chastang JF David S Kelleher C. The contribution of occupational factors to social inequalities in health: Findings from the national French SUMER survey. Soc Sci Med. (2008) 67:1870–81. 10.1016/j.socscimed.2008.09.007 18851892

[4] Devillanova C Raitano M Struffolino E. Longitudinal employment trajectories and health in middle life: Insights from linked administrative and survey data. Demogr Res. (2019) 40:1375–412. 10.4054/DemRes.2019.40.47

[5] Murcia M Chastang JF Cohidon C Niedhammer I. Samotrace study group. Contribution of occupational factors to social inequalities in self-reported health among French employees. Int Arch Occup Environ Health. (2013) 86:541–52. 10.1007/s00420-012-0784-2 22678750

[6] Kjellsson S. Accumulated occupational class and self-rated health. Can information on previous experience of class further our understanding of the social gradient in health? Soc Sci Med. (2013) 81:26–33. 10.1016/j.socscimed.2013.01.006 23422057

[7] Bambra C Lunau T Van der Wel KA Eikemo TA Dragano N. Work, health, and welfare: the association between working conditions, welfare states, and self-reported general health in Europe. Int J Health Serv. (2014) 44:113–36. 10.2190/HS.44.1.g 24684087

[8] Shockey TM Zack M Sussell A. Health-related quality of life among US workers: Variability across occupation groups. Am J Public Health. (2017) 107:1316–23. 10.2105/AJPH.2017.303840 28640675 PMC5508147

[9] Kong KA Khang YH Cho HJ Jang SM Jung-Choi K. Neo-marxian social class inequalities in self-rated health among the employed in South Korea: The role of material, behavioral, psychosocial, and workplace environmental factors. BMC Public Health. (2017) 17:345. 10.1186/s12889-017-4269-9 28427359 PMC5397726

[10] Kaleta D Makowiec-Dabrowska T Jegier A. Employment status and self rated health. Int J Occup Med Environ Health. (2008) 21:227–36. 10.2478/v10001-008-0023-y 18842579

[11] de Oliveira TL Griep RH Guimarães JN Giatti L Chor D da Fonseca MD. Brazilian longitudinal study of adult health (ELSA-Brasil): socio-occupational class as an effect modifier for the relationship between adiposity measures and self-rated health. BMC Public Health. (1944) 19:734. 10.1186/s12889-019-7072-y 31185963 PMC6560819

[12] Granström F Molarius A Garvin P Elo S Feldman I Kristenson M. Exploring trends in and determinants of educational inequalities in self-rated health. Scand J Public Health. (2015) 43:677–86. 10.1177/1403494815592271 26138729

[13] Franks P Gold MR Fiscella K. Sociodemographics, self-rated health, and mortality in the US. Soc Sci Med. (2003) 56:2505–14. 10.1016/S0277-9536(02)00281-2 12742613

[14] Mirowsky J Ross CE. Education and self-rated health: cumulative advantage and its rising importance. Res Aging. (2008) 30:93–122. 10.1177/016402750730964929337605

[15] Mcfadden E Luben R Bingham S Wareham N Kinmonth A Khaw K. Social inequalities in self-rated health by age: Cross-sectional study of 22 457 middle-aged men and women. BMC Public Health. (2008) 8:230. 10.1186/1471-2458-8-230 18611263 PMC2491612

[16] Rodrigues CG Maia AG. How does social position influence self-reported health status? A comparative analysis between 1998 and 2003. Cad Saude Publica. (2010) 26:762–74. 10.1590/S0102-311X2010000400018 20512216

[17] Borg V Kristensen TS Burr H. Work environment and changes in self-rated health: a five year follow-up study. Stress Health. (2015) 16:37–47. 10.1002/(SICI)1099-1700(200001)16:1<37::AID-SMI830>3.0.CO;2-O

[18] Cho SS Ju YS Paek D Kim H Jung-Choi K. The combined effect of long working hours and low job control on self-rated health: an interaction analysis. J Occup Environ Med. (2018) 60:475–80. 10.1097/JOM.0000000000001241 29200187 PMC5959214

[19] Ang YY. Counting Cadres: A comparative view of the size of China's public employment. China Q. (2012) 211:676–96. 10.1017/S0305741012000884

[20] Marchand A Demers A Durand P. Social structures, agent personality and workers' mental health: a longitudinal analysis of the specific role of occupation and of workplace constraints-resources on psychological distress in the Canadian workforce. Hum Relat. (2006) 59:875–901. 10.1177/0018726706067595

[21] Rosenbaum PR Rubin DB. The central role of the propensity score in observational studies for causal effects. Biometrika. (1983) 70:41–55. 10.1093/biomet/70.1.41

[22] Dehejia RH Wahba S. Causal effects in nonexperimental studies: reevaluating the evaluation of training programs. J Am Stat Assoc. (1999) 94:1053–62. 10.1080/01621459.1999.10473858

[23] Leonard C Fanning N Attwood J Buckley M. The effect of fatigue, sleep deprivation and onerous working hours on the physical and mental wellbeing of pre-registration house officers. Ir J Med Sci. (1998) 167:22–5. 10.1007/BF02937548 9540294

[24] Zhang X Wang Z Li T. The current status of occupational health in China. Environ Health Prev Med. (2010) 15:263–70. 10.1007/s12199-010-0145-2 21432554 PMC2921040

[25] Siegrist J. Adverse health effects of high-effort/low-reward conditions. J Occup Health Psychol. (1996) 1:27–41. 10.1037/1076-8998.1.1.27 9547031

[26] Schütte S Chastang JF Parent-Thirion A Vermeylen G Niedhammer I. Association between socio-demographic, psychosocial, material and occupational factors and self-reported health among workers in Europe. J Public Health. (2014) 36:194–204. 10.1093/pubmed/fdt050 23695703

[27] Silver SR Alarcon WA Li J. Incident chronic obstructive pulmonary disease associated with occupation, industry, and workplace exposures in the Health and Retirement Study. Am J Ind Med. (2021) 64:26–38. 10.1002/ajim.23196 33124723 PMC7736507

[28] Aldabe B Anderson R Lyly-Yrjänäinen M Parent-Thirion A Vermeylen G Kelleher CC. Contribution of material, occupational, and psychosocial factors in the explanation of social inequalities in health in 28 countries in Europe. J Epidemiol Community Health. (2011) 65:1123–31. 10.1136/jech.2009.102517 20584725 PMC3678208

[29] Winkleby MA Jatulis DE Frank E Fortmann SP. Socioeconomic status and health: How education, income, and occupation contribute to risk factors for cardiovascular disease. Am J Public Health. (1992) 82:816–20. 10.2105/AJPH.82.6.816 1585961 PMC1694190

[30] Moran CC. Stress and emergency work experience: a non-linear relationship. Disaster Prev Manag. (1998) 7:38–46. 10.1108/09653569810206271

[31] Gove WR Ortega ST Style CB. The maturational and role perspectives on aging and self through the adult years: an empirical evaluation. Am J Sociol. (1989) 94:1117–45. 10.1086/229113

[32] Mirowsky J Ross CE. Age and the effect of economic hardship on depression. J Health Soc Behav. (2001) 42:132–50. 10.2307/3090174 11467249

[33] Schieman S Gundy KV Taylor J. The relationship between age and depressive symptoms: a test of competing explanatory and suppression influences. J Aging Health. (2002) 14:260–85. 10.1177/089826430201400205 11995743

[34] Jones G. Higher education can enhance career advancement. Can Occup Saf. (2017) 55:10.

[35] Lynch JW Kaplan GA Salonen JT. Why do poor people behave poorly? Variation in adult health behaviours and psychosocial characteristics by stages of the socioeconomic lifecourse. Soc Sci Med. (1997) 44:809–19. 10.1016/S0277-9536(96)00191-8 9080564

[36] Dembe AE Erickson JB Delbos RG Banks SM. The impact of overtime and long work hours on occupational injuries and illnesses: new evidence from the United States. Occup Environ Med. (2005) 62:588–97. 10.1136/oem.2004.016667 16109814 PMC1741083

[37] Nakata A. Investigating the associations between work hours, sleep status, and self-reported health among full-time employees. Int J Public Health. (2012) 57:403–11. 10.1007/s00038-011-0242-z 21384225

[38] Cho SS Ki M Kim KH Ju YS Paek D Lee W. Working hours and self-rated health over 7 years: Gender differences in a Korean longitudinal study. BMC Public Health. (2015) 15:1287. 10.1186/s12889-015-2641-1 26701111 PMC4690406

[39] Dembe AE. Ethical issues relating to the health effects of long working hours. J Bus Ethics. (2009) 84(suppl. 2):195–208. 10.1007/s10551-008-9700-9

[40] Sato K Kuroda S Owan H. Mental health effects of long work hours, night and weekend work, and short rest periods. Soc Sci Med. (2020) 246:112774. 10.1016/j.socscimed.2019.112774 31923837

[41] Nakanishi N Yoshida H Nagano K Kawashimo H Nakamura K Tatara K. Long working hours and risk for hypertension in Japanese male white collar workers. J Epidemiol Community Health. (2001) 55:316–22. 10.1136/jech.55.5.316 11297649 PMC1731895

[42] Yang H Schnall PL Jauregui M Su TC Baker D. Work hours and self-reported hypertension among working people in California. Hypertension. (2006) 48:744–50. 10.1161/01.HYP.0000238327.41911.52 16940208

[43] Popham F Mitchell R. Leisure time exercise and personal circumstances in the working age population: longitudinal analysis of the British household panel survey. J Epidemiol Community Health. (2006) 60:270–4. 10.1136/jech.2005.041194 16476760 PMC2465565

[44] Neel R Lassetter B. Growing fixed with age: lay theories of malleability are target age-specific. Pers Soc Psychol Bull. (2015) 41:1505–22. 10.1177/0146167215600529 26351273

